# Differential Effects of TNF (TNFSF2) and IFN-γ on Intestinal Epithelial Cell Morphogenesis and Barrier Function in Three-Dimensional Culture

**DOI:** 10.1371/journal.pone.0022967

**Published:** 2011-08-11

**Authors:** Kati Juuti-Uusitalo, Leon J. Klunder, Klaas A. Sjollema, Katarina Mackovicova, Ryuichi Ohgaki, Dick Hoekstra, Jan Dekker, Sven C. D. van IJzendoorn

**Affiliations:** 1 Top Institute Food and Nutrition, Wageningen, The Netherlands; 2 Department of Cell Biology, Section of Membrane Cell Biology, University Medical Center Groningen, University of Groningen, Groningen, The Netherlands; 3 Department of Cell Biology, Section of Molecular Imaging and Electron Microscopy, University Medical Center Groningen, University of Groningen, Groningen, The Netherlands; 4 UMCG Microscopy and Imaging Center (UMIC), University Medical Center Groningen, University of Groningen, Groningen, The Netherlands; Emory University, United States of America

## Abstract

**Background:**

The cytokines TNF (TNFSF2) and IFNγ are important mediators of inflammatory bowel diseases and contribute to enhanced intestinal epithelial permeability by stimulating apoptosis and/or disrupting tight junctions. Apoptosis and tight junctions are also important for epithelial tissue morphogenesis, but the effect of TNF and IFNγ on the process of intestinal epithelial morphogenesis is unknown.

**Methods/Principal Findings:**

We have employed a three-dimensional cell culture system, reproducing in vivo-like multicellular organization of intestinal epithelial cells, to study the effect of TNF on intestinal epithelial morphogenesis and permeability. We show that human intestinal epithelial cells in three-dimensional culture assembled into luminal spheres consisting of a single layer of cells with structural, internal, and planar cell polarity. Exposure of preformed luminal spheres to TNF or IFNγ enhanced paracellular permeability, but via distinctive mechanisms. Thus, while both TNF and IFNγ, albeit in a distinguishable manner, induced the displacement of selected tight junction proteins, only TNF increased paracellular permeability via caspase-driven apoptosis and cell shedding. Infliximab and adalumimab inhibited these effects of TNF. Moreover, we demonstrate that TNF via its stimulatory effect on apoptosis fundamentally alters the process of intestinal epithelial morphogenesis, which contributes to the *de novo* generation of intestinal epithelial monolayers with increased permeability. Also IFNγ contributes to the *de novo* formation of monolayers with increased permeability, but in a manner that does not involve apoptosis.

**Conclusions:**

Our study provides an optimized 3D model system for the integrated analysis of (real-time) intestinal epithelial paracellular permeability and morphogenesis, and reveals apoptosis as a pivotal mechanism underlying the enhanced permeability and altered morphogenesis in response to TNF, but not IFNγ.

## Introduction

The intestinal epithelium is a selectively permeable single-cell layer, which is subject to continuous renewal. This includes progenitor proliferation, directional migration of epithelial cells from the crypt region and, ultimately, cell death and shedding [1]. This morphogenic process is tightly controlled in time and space to ensure maintenance of the characteristic monolayer-type organization and, consequently, an adequate barrier function. Inflammatory bowel diseases such as Crohn's disease are characterized by mucosal and epithelial injury and barrier abnormalities, including changes in epithelial tight junctions, mucosal lesions, epithelial restoration failure, and changed functionality of the epithelial cells, which are correlated with immune deregulation [2]. Little is known about the molecular events that cause intestinal epithelial remodelling during inflammatory processes. The excessive secretion of proinflammatory cytokines plays an integral role in the pathogenesis of inflammatory diseases [3], [4]. For instance, Crohn's disease is associated with hyperactivation of T helper 1 (Th1) cells with abundant secretion of interferon (IFN)γ and tumor necrosis factor (TNF). These cytokines mediate a variety of biological effects that potentiate the immune response, which can lead to, e.g., oedema in the lamina propria and consequent breaks in the epithelial monolayer [5]. In addition, these cytokines can directly target intestinal epithelial cells to elicit signalling pathways that stimulate apoptosis and/or inhibit the function of tight junctions, both of which may result in reduced epithelial integrity [6]–[8]. Treatment of patients with active Crohn's disease with the TNF inhibitor infliximab has been reported to reduce gut inflammation and largely restore the gut barrier, underscoring the important role of TNF in IBD [8], [9].

While regulated apoptosis and cell-cell adhesions are crucial to maintain the barrier integrity of existing monolayers, apoptosis and cell-cell adhesion are also important for proper epithelial morphogenesis, i.e. the assembly of intestinal epithelial cells into a stable, single-layered polarized tissue [10]. Epithelial morphogenesis is crucial to maintain the integrity of a constitutively developing and differentiating tissue [11], such as the gut epithelium. Epithelial morphogenesis requires the establishment of an apical-basal axis of polarity and the formation of apical, lateral and basal cell surface domains with the appropriate adhesive junctions and, in concert with this, a remodeling of the cytoskeleton and polarized vesicular transport to secure these domains [12], [13]. It furthermore requires a planar orientation of cell division [14]–[16]. This ensures that newly formed epithelial daughter cells stay within the monolayer, thereby preserving the integrity of the monolayer during cell division [14]–[16]. Together, these events allow cells to generate a stable cell monolayer that is able to functionally cope with distinct extracellular environments (i.e. gut lumen versus body tissue and fluids). Whereas the effects of proinflammatory Th1 cytokines like TNF and IFNγ on existing epithelial monolayers have been studied in detail, it is not known whether exposure of intestinal epithelial cells to such cytokines interferes with the process of epithelial morphogenesis and, as such, may perturb the functional differentiation of a regenerative intestinal epithelium.

In this study we have investigated the involvement of TNF and IFNγ in epithelial morphogenesis employing a three-dimensional (3D) intestinal epithelial cell culture system. 3D epithelial culture systems allow key events in the life cycle of intestinal epithelial cells, such as proliferation, differentiation, apoptosis and migration, to be controlled in concert by organizing principles that are determined by the spatial context of the cells. 3D culture systems, therefore, mimic essential aspects of the *in vivo* organisation of epithelial cells of various origins [17]–[20]. Epithelial cells cultured in 3D matrix self-assemble into polarized monolayers that separate central apical lumens from a basal environment containing extracellular matrix. We have further characterized and implemented a previously reported 3D intestinal epithelial model system [16], [21] for the quantitative analysis of real-time epithelial permeability changes, and show that TNF perturbs and, by stimulating apoptosis, alters the process of gut epithelial morphogenesis.

## Materials and Methods

### Cell culture

Human intestinal epithelial T84 cells (ATCC Rockville, MD/USA) were cultured in 5% CO_2_ at 37°C in Dulbecco's modified Eagle medium (DMEM)/Ham's F-12 (1∶1) medium (Gibco-BRL, Paisley/Scotland), supplemented with 10% heat-inactivated fetal calf serum (Gibco-BRL) and antibiotics (500 IU/mL penicillin/100 µl/ml streptomycin, Gibco-BRL). Human colorectal carcinoma Caco-2 cells (15) were cultured in DMEM with 4.5 g/L glucose and supplemented with 10% heat-inactivated fetal calf serum (Gibco-BRL) and antibiotics (500 IU/mL penicillin/100 µl/ml streptomycin, Gibco-BRL). Cells were seeded at 500 cells/µl in 35% (v/v) Matrigel™ (BD Biosciences, MA/USA) and cell culture medium, according the manufacturer's instructions and essentially as described elsewhere [15]. Cells were cultured for 7 days and medium was refreshed every 2–3 days. For immunofluorescence investigation cells were cultured in Lab-Tec Chamber Slides (Nunc, Rochester, NY/USA), and for live-cell barrier integrity assessment in MatTec glass-bottom dishes (MatTec, Ashland, MA/USA), and for electron microscopy experiments in 24-well plates (Nunc, Rochester, NY/USA).

### Immunofluorescence microscopy

3D cell cultures were fixed with 4% paraformaldehyde at 37°C for 1 h, permeabilized with 0.1% Triton-X100 (Sigma-Aldrich, Selze/Germany) at room temperature for 45 minutes and washed with Hank's balanced salt solution (HBSS). Following blocking in 3% BSA in HBSS at 37°C, spheres were immunolabeled with mouse anti-CD10 (1∶200, gift from dr. Karrenbeld, UMCG/Netherlands), mouse anti-gp135 (1∶150, gift from dr. Mostov, UCSF/USA), mouse anti-β-catenin (1∶250 BD Transduction Laboratories, CA/USA) rabbit anti-ZO-1 (1∶100; Invitrogen, Carlsbad/USA), mouse anti-occludin (Zymed/Invitrogen, USA), rabbit anti-NHE8( 1∶100, gift from dr. Kanazawa, Osaka University/Japan), rabbit anti-giantin (1∶500, Covance/USA), mouse anti-β1-integrin (1∶100, hybridoma bank), rabbit anti-laminin1/2 (1∶100, Abcam, Cambridge/UK), rabbit anti-claudin-1 (Invitrogen, USA), and/or mouse anti-acetylated α-tubulin (Abcam). All primary antibody incubations were performed at 37°C for 1.5 h. Samples were washed with HBSS and labeled with secondary Alexa-Fluor488 conjugated goat anti-rabbit or Alexa-Fluor488 goat anti-mouse antibody (1∶1500; Molecular Probes, Leiden/The Netherlands). Filamentous actin was labeled with TRITC-phalloidin (1∶1500 Sigma-Aldrich). In double labelings, Alexa-Fluor546 conjugated goat anti-rabbit or goat anti-mouse was used as secondary antibody. Labeled cells were mounted with DAPI-containing VectaShield Mounting Medium (Vector Laboratories, CA/USA).

For evaluating the distribution profile of cell-cell adhesion proteins along the lateral plasma membrane, fluorescence intensity data from the green and red channels along the lateral plasma membranes of cells in a confocal section taken from the middle of a sphere were collected with ImageJ software, exported to MSExcell and plotted. Each analysis included at least 5 spheres with at least 7 lateral plasma membrane domains per sphere.

Apoptotic index was determined with ApopTag Red *in situ* Apoptosis detection kit (Chemicon International, CA/USA) according to the manufacturer's instructions. Samples were analyzed with an Olympus Provis AX70 fluorescence microscope or witha Leica TCS SP2 AOBS confocal microscope. Stack deconvolution and 3D reconstruction were done using Huygens (SVI, Hilversum/The Netherlands) IMARIS software (Bitplane).

### Electron microscopy

3D cell cultures were rinsed with 0.1 M cacodylate buffer (pH 7.4), fixed with 2% glutaraldehyde in 0.1 M cacodylate buffer (room temperature; 2 h), and washed in 0.1 M cacodylate buffer. Cells were incubated in 1% OsO4/0.5% K4Fe(CN)6 in 0.1 M cacodylate buffer on ice for 2 h. Samples were washed with water and dehydrated in series of ethanol After dehydration, samples were incubated in 1∶1 mixture of 100% ethanol∶EPON for 1 h at room temperature, then in 2∶1 mixture of 100% ethanol∶EPON for 1 h in room temperature, and in 100% EPON at room temperature for 2 h. Samples were incubated in vacuum at room temperature for 1 h, after repeated refreshing of 100% EPON for 2 h vacuum at 45°C and finally over night at 60°C. Sections were made using LKB Ultramicrotome. Images were taken on a Phillips CM100 transmission electron microscope.

### Determination of epithelial barrier integrity

Cells were cultured in Matrigel for 5 days, after which 10 ng/ml TNF or 100 IU/ml IFNγ (Peprotec, London, UK), culture medium (control) and/or the pancaspase inhibitor Q-VD-OPH (50 µM, Calbiochem, CA/USA), infliximab or adalumimab (1 µg/ml; gift from dr. G. Dijkstra, University Medical Center Groningen, The Netherlands) was added for another 48 h. Spheres were then exposed to FITC-dextran of 4 kDa (FD4, Sigma-Aldrich) for 1 h at 37°C. As a positive control, spheres were treated with EGTA (2 mM, Sigma-Aldrich) to disrupt tight junctions. Concentrations and incubation times were similar as those used by other groups in previously publications. Live cell imaging was performed on a Leica Solamere confocal microscope in a humidified incubator at 37°C. Images were taken every 5 min. Changes in barrier integrity were determined from live-cell imaging pictures. Fluorescence intensity in luminal side (FL) and basal medium are expressed as ratio FL/FB where the maximal permeability (100%) was determined following EGTA treatment.

### Epithelial morphogenesis studies

T84 cells were cultured in medium (control), supplemented or not with 10 ng/ml TNF, 100 IU/ml IFNγ and/or the pancaspase inhibitor Q-VD-OPH (50 µM, Calbiochem, CA/USA) for up to 72 h. Spheres were examined as described above every 24 h after seeding.

### Sodium dodecyl sulfate polyacrylamide gel electrophoresis and Western blotting

Cells were lysed in lysis buffer (PBS containing 1% NP-40 and protease inhibitors: 1 µg/ml aprotinin, 100 µM benzamidine, 0.5 µg/ml leupeptin, and 1 µg/ml pepstatin A). After incubation on ice for 15 min, cells were homogenized in a syringe with a 27-gauge needle (20 strokes) and centrifuged for 10 min at 13,200×*g* at 4°C. The supernatant fraction was collected and the protein concentration was determined with a bicinchoninic acid (BCA) protein assay. 60 micrograms of protein per lane was separated in a SDS-polyacrylamide (12.5% (w/v) gel. Proteins were transferred (semi-dry) onto PVDF membranes. For the detection of rabbit polyclonal claudin-1 (Invitrogen) or mouse monoclonal β-tubulin (Millipore) antibody reactivity, an ODYSSEY infrared imaging system (LI-COR Biosciences, Westburg BV, the Netherlands) was used according to the manufacturer's instruction.

## Results

### Intestinal epithelial cells in 3D culture form polarized monolayers

**Figure 1:**
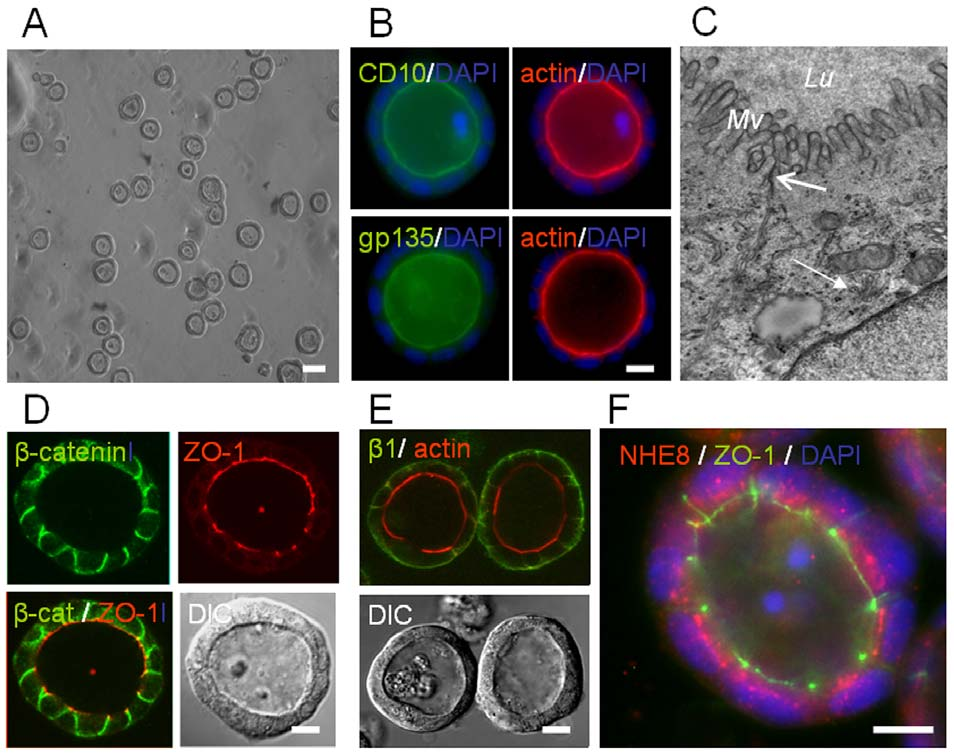
T84 cells in 3D culture develop a polarized monolayer that separates an apical and basal extracellular environment. A) Low magnification phase contrast image of 7 days-old T84 in 3D culture. B) CD10 and gp135 (podocalyxin) are exclusively expressed at the actin filament-rich luminal cell surface of the spheres. C) Transmission electron microscopical cross section of a hollow sphere. Note microvilli (*Mv*) facing the lumen (*Lu*). Electron dense tight junctions and an apically orientated Golgi apparatus are indicated by the open and closed arrow, respectively. D) β-catenin and ZO-1 localize along the lateral surface and at the apical-most apex of the lateral surface, respectively. E) The β-1 integrin receptor localizes to the basolateral domain of the luminal sphere formed by T84 cells. Corresponding DIC image is depicted in the lower panel. F) Golgi-associated NHE8 is orientated towards the apical surface. ZO-1 is labeled in green. In B,D-F, cell nuclei are stained with DAPI (blue). Bars: 40 µm (A), 10 µm (B, D–F).

Human T84 cells, widely used to study various aspects of intestinal epithelial cell biology, were cultured in Matrigel™. The cells readily (i.e. starting within 1 day) formed hollow spheres of uniform size (30–45 µm diameter) consisting of a single layer of 25–40 cells (visualized by DAPI-stained nuclei) surrounding a central lumen (Figure 1A,B). Proteins that are typically expressed at the intestinal brush border, the metalloendopeptidase CD10, podocalyxin (gp135), and filamentous actin, predominantly localized to the cell surfaces lining the lumens of the spheres (Figure 1B), indicating that the sphere lumen was representative of the gut lumen. The adherens junction-associated protein β-catenin predominantly localized to sites of cell-cell contact (Figure 1D). The tight junction-associated protein ZO-1 was organized in a belt around the cells' apex (Figure 1D). A 3D graphical reconstruction of deconvoluted confocal image stacks taken from a sphere is shown in Movie S1, and demonstrate the typical organization of tight junctions and adherens junctions along the lateral cell surface. Transmission electron microscopy confirmed that the cells developed microvilli-rich brush border domains facing the lumen, and electron-dense tight junctions could be readily distinguished between cells (Figure 1C). β1 integrin localized to the basal and lower lateral domains of the cells (Figure 1E), just as it does *in vivo*. Finally, the Golgi apparatus was positioned in the supranuclear region of the cells facing the apical brush border domain, evidenced by transmission electron microscopy (Figure 1C), and fluorescence microscopy (Figure 1F). These data demonstrate that T84 cells assembled into a monolayer with structural, internal, and planar cell polarity, as displayed by enterocytes in the intestine. Also Caco-2 cells, another human intestinal epithelial model cell line formed polarized luminal spheres (Figure S1, B,C), although these cells displayed considerable heterogeneity with regard to sphere morphology (Figure S1,A).

### TNF and IFNγ increase the permeability of intestinal epithelial monolayers in 3D culture

**Figure 2:**
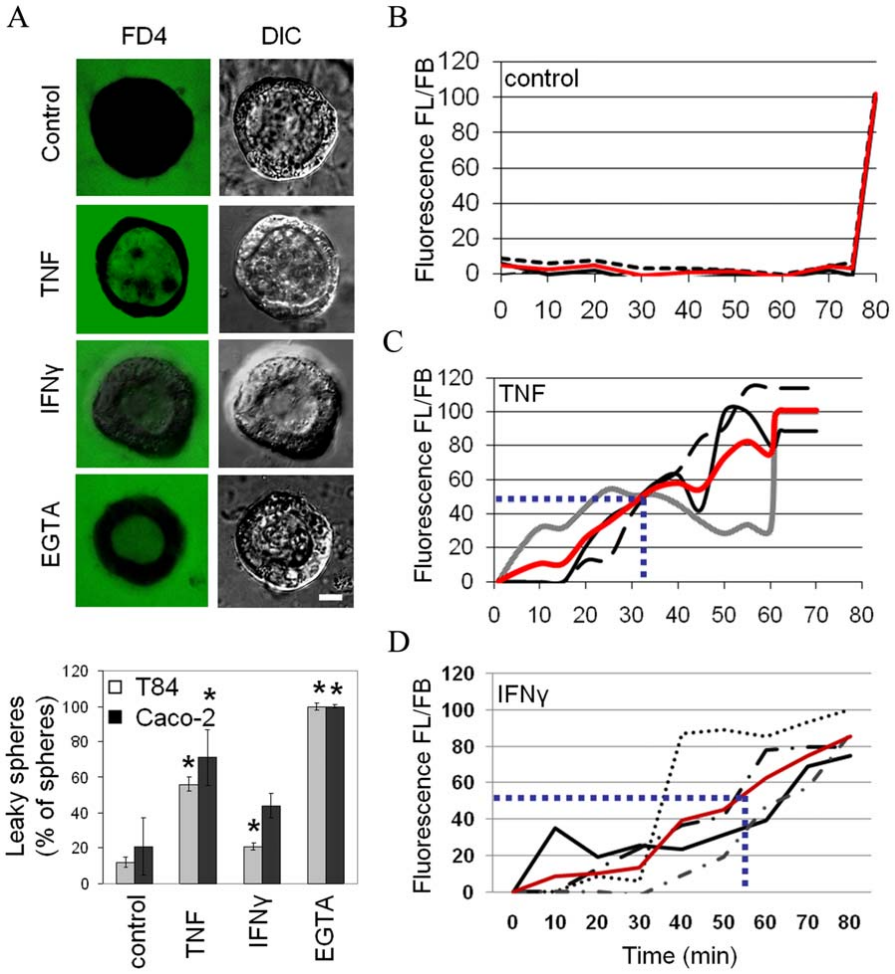
Effect of TNF and IFNγ on paracellular permeability in 3D T84 and Caco-2luminal spheres. A) Five days-old spheres of T84 cells were treated or not treated with TNF for 48 h and exposed to FD4 at 37°C for 1 h and fixed. Occasionally, EGTA was included during FD4 treatment. Note the appearance of FD4 in the apical lumens of TNF-, IFNγ- and EGTA-exposed, but not untreated spheres (left column). Corresponding DIC images are depicted in the right column. The graph depicts the percentage of total hollow T84 spheres containing FD4 in their lumen. Asterisks indicate statistical significance in changes in percentages of leaky spheres T84 and Caco-2 relative to their respective controls in a Student's t-test (p<0.05). B–D) Five days-old spheres of T84 cells were treated or not treated with TNF or IFNγ for 48 h and exposed to FD4 at 37°C for 1 h on a live cells imaging microscope. After 1 h EGTA was added (positive control, i.e. 100% leaky spheres). At each time point, the ratio of FD4 fluorescence in the luminal contents (FL) over the FD4 fluorescence in the basolateral medium (FB), as an indication of paracellular permeability, was determined. Paracellular permeability was set to zero at the start of the measurement in non-treated control cells and set to 100% following EGTA exposure. Three control and TNF- or IFNγ-treated T84 spheres are depicted (panel B, C and D respectively). The red line indicates the average.

**Figure 3:**
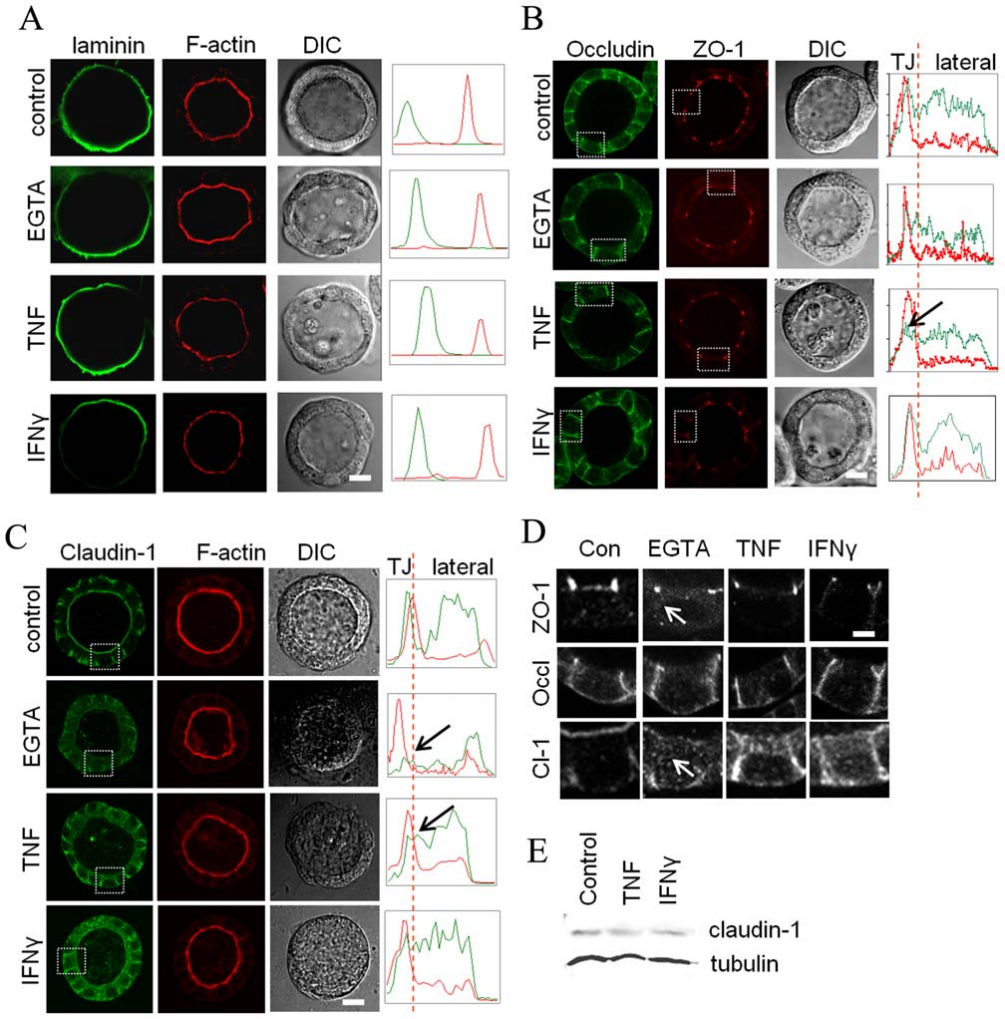
Effect of TNF and IFNγ on subcellular distribution of tight junction proteins. A–C) Five days-old spheres were treated or not with TNF and immunolabeled with antibodies against laminin1/2 (A, left column), occludin (B, left column), ZO-1 (B, middle column), or claudin-1 (C, left column). In panel A (middle column) cells were stained with TRITC-phalloidin to visualize the actin filament-rich apical surface. Corresponding DIC images are depicted in the right columns of panels A, B, and C. Boxed areas in panels B (left and middle column) and C (left column) correspond to the enlarged images in panel D. Bars 10 µm. Graphs in panels A–C show the distribution profiles of tight junction proteins (green and red channels) from the apex to the basal side of the lateral plasma membranes. Arrows point to the absence of a peak expression at the TJ area when compared to control cells. D) Enlarged images corresponding to the boxed areas indicated in panels B and C. The arrow points to cytoplasmic ZO-1 or claudin-1 staining in EGTA-treated spheres. Bar 5 µm. E. Western blot showing the expression level of claudin-1 in control cells and cells treated with TNF or IFNγ. Tubulin was used as an internal control for loaded cell proteins.

We next investigated the effects of TNF and IFNγ on the integrity and permeability of pre-formed 3D intestinal epithelial monolayers. T84 or Caco-2 cells were cultured for 5 days after which cells had assembled into uniform hollow spheres. Spheres were then treated with or without TNF (10 ng/ml) or IFNγ (100 IU/ml) for 48 h and exposed to FITC-labeled dextran of 4 kDa (FD4). FD4 was efficiently excluded from the apical lumen of control, single lumen-containing spheres indicating that the cells in single lumen-containing spheres had formed tight monolayers (Figure 2A and Figure S1,C). In contrast, FD4 fluorescence was readily observed in the apical lumen of 47±3% (p<0.01) of TNF-treated T84 spheres and of 21±2% (p<0.05) of IFNγ-treated T84 spheres (Figure 2A). Similarly, FD4 fluorescence was readily observed in the apical lumen of 54±2% (p<0.05) of TNF-treated and 18±4% (p = 0.07) of IFNγ-treated single lumen-containing Caco-2 spheres (Figure 2A). As a positive control, control spheres were treated with the Ca^2+^-chelator EGTA (2 mM), which disrupts tight junctions. Indeed, FD4 rapidly equilibrates from the basolateral side to the apical lumen of all EGTA-treated control spheres (Figure 2A and Figure S1,C). Overall sphere morphology was maintained after cytokine treatment (Figure 2A and Figure 3), and immunolabeling of the basal extracellular matrix protein laminin1/2 revealed that it maintained its exclusive localization at the basal surface of all spheres (Figure 3A). The organisation of filamentous actin at the apical poles in the cells of the spheres after TNF or IFNγ treatment was unaltered (Figure 3A). TNF and IFNγ did not visibly alter the distribution of the tight junction-associated protein ZO-1. By contrast, TNF and IFNγ did alter the distribution of other tight junction proteins, albeit in a distinct manner. Thus, whereas occludin and claudin-1 intensities in untreated spheres peaked at the cell apices and was additionally distributed along the lateral surfaces, in TNF-treated but not in IFNγ-treated spheres, occludin and claudin-1 failed to peak at the cell apices (Figure 3B, C). Both TNF and IFNγ induced the appearance of claudin-1, but not occludin or ZO-1, in intracellular puncta (Figure 3D). No changes in the total expression of claudin-1 was observed between TNF or IFNγ-treated cells and control cells (Figure 3E).

Paracellular permeability to FD4 was also investigated in T84 spheres using live cell fluorescence microscopy. Control, TNF- or IFNγ-treated spheres were exposed to FD4 in the basolateral medium for 90 min. At different time points the fluorescence intensity from FD4 in the basal and apical/luminal space was measured and expressed as the ratio of fluorescence in the luminal compartment (FL) over that in the basal compartment (FB). As shown in figure 2B, all non-treated spheres that were measured excluded FD4 from the apical lumen, evidenced by a very low FL/FB ratio, and displayed an increase in permeability following EGTA addition. Interestingly, TNF- and IFNγ-treated spheres (3 tracings of individual spheres are depicted) all showed increased paracellular permeability, but with variable rate and to a variable extent (Figure 2C,D). Paracellular leakage of FD4 in TNF-treated cells occurred at a higher rate when compared to IFNγ-treated cells (Figure 2C,D). Thus, the average time to reach half maximum leakage in response to TNF or IFNγ was 32 or 56 min, respectively. (Figure 2C, D, blue lines), which seems consistent with the higher percentage of leaky spheres in response to TNF (Figure 2A). These data demonstrate that both TNF and IFNγ increase the paracellular permeability of intestinal epithelial monolayers in 3D cultures to small molecular weight molecules, albeit with distinguishable kinetics and with distinguishable effects on the subcellular distribution of tight junction proteins.

### TNF, but not IFNγ, increases the mitotic index and stimulates apoptosis of intestinal monolayers in 3D culture

**Figure 4:**
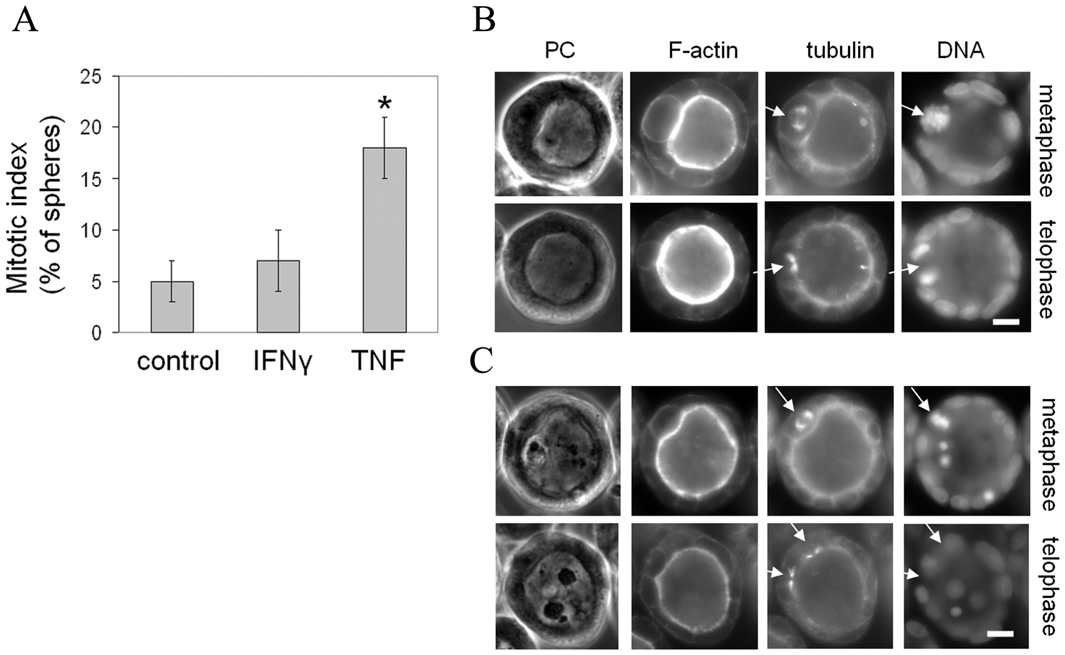
TNF but not IFNγ increases the mitotic index in 3D T84 cell cultures. Five days-old spheres were treated (A, C) or not treated (A, B) with TNF or IFNγ for 48 h, fixed and immunolabeled with antibodies against α-tubulin (B, C; middle-right column) to visualize the mitotic spindle and stained with the DNA-binding dye DAPI to visualize prophase and metaphase cells (B, C; right column). Cells were also stained with TRITC-phalloidin to visualize the apical surface (middle-left column). Corresponding phase-contrast (PC) images are depicted in the left column (B, C). The percentage of spheres containing mitotic cells is depicted in A, where the asterisk indicates statistical significance in a Student's t-test (p<0.05). Arrows indicate mitotic spindles. Bars 10 µm.

The number of spheres containing mitotic cells increased from 5% to 18% following treatment with TNF, as determined by the presence of mitotic DNA figures and tubulin-marked mitotic spindles (Figure 4A). In contrast to TNF-treated cells, no increase in the mitotic index was observed in IFNγ-treated cells (Figure 4A). The remaining 82% of the TNF-treated spheres did not reveal any mitotic cells. In mitotic cells, we found that the orientation of the mitotic spindle in TNF-treated cells was predominantly orientated within the plane of the monolayer, i.e. parallel to the apical surface and lumen (Figure 4C, arrows). This was comparable to the orientation of the mitotic spindle in non-treated spheres (Figure 4B) and in agreement with reports from 2D cell cultures [16] and intestinal biopsies [15]. Accordingly, cell multilayering, which could have been a result of disorientated cell division [16], was not observed.

**Figure 5:**
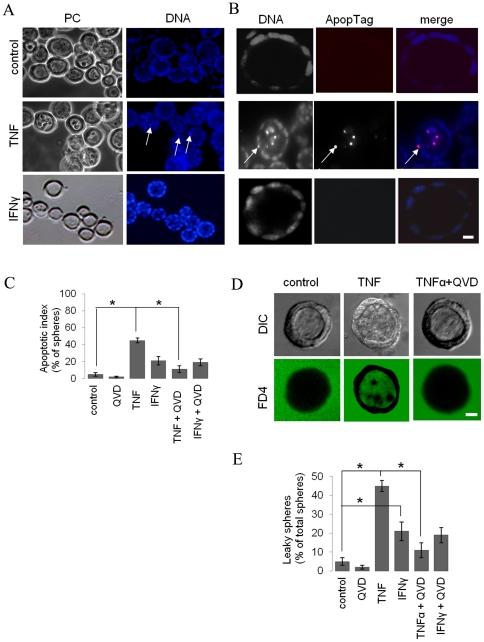
TNF, but not IFNγ, stimulates apoptosis. A) 7 days-old T84 spheres shown by phase contrast microscopy (left column) were stained with DAPI to visualize the DNA (right column). Note the appearance of bright micronuclei in the monolayer and lumen of TNF-, but not IFNγ-treated spheres (arrows). B) Control, TNF- and IFNγ-treated spheres were immunolabeled with ApopTag and stained with the DNA binding dye DAPI. Note that the bright DAPI-stained micronuclei are positive for ApopTag (purple colour in ‘merge’). C) The apoptotic index, expressed as the percentage of all luminal spheres that contain ApopTag-positive cells in the presence/absence of the apoptosis inhibitor QVD is depicted in the graph. D) Five days-old spheres were treated or not treated with TNF and/or the caspase inhibitor QVD for 48 h and exposed to FD4 at 37°C for 1 h and fixed. E) Quantification of data as displayed in panel D. Note that the luminal appearance of FD4 in TNF-, but not IFNγ-treated spheres is counteracted by the co-incubation with QVD. Asterisks indicate statistical significance in a Student's t-test (p<0.05). Bars indicate 10 µm.

TNF, but not IFNγ, stimulated the accumulation of DAPI-positive material in the apical lumen of T84 spheres that resembled condensed and/or fragmented nuclei (Figure 5A), indicative of apoptosis. To verify that these represented apoptotic cells, we stained apoptotic cells *in situ* by labeling and detecting DNA strand breaks by the TUNEL method. Apoptotic cells positively correlated with condensed DAPI-stained nuclei in spheres that were treated with TNF (Figure 5B). Notably, apoptotic cells resided in the apical lumen as well as in the monolayer of TNF-treated cells (Figure 5B, arrow). Less than 20% of non-treated control or IFNγ-treated spheres contained apoptotic cells, whereas ∼75% of TNF-treated spheres contained apoptotic cells (Figure 5B).

### TNF-stimulated permeability is secondary to TNF-stimulated apoptosis

To determine whether TNF-stimulated apoptosis is involved in the TNF-stimulated permeability of the luminal spheres, 5 day-old (post-seeding) spheres were treated with or without TNF and/or the caspase-inhibitor Q-VD-OPH (QVD). Treatment with QVD prevented apoptosis in TNF-treated cells, resulting in spheres with virtually no luminal cellular debris (Figure 5D). We next examined the permeability of the luminal spheres by including FD4 in the medium. Whereas TNF-treated spheres showed a pronounced accumulation of FD4 in their apical lumens (Figure 2A), co-treatment with QVD reduced the number of spheres that had leaked FD4 into their apical lumens (Figure 5D,E). These data indicate that TNF-stimulated apoptosis, at least in part, mediated the TNF-stimulated permeability of the intestinal luminal sphere. QVD did not prevent the enhanced paracellular leakage of FD4 in IFNγ-treated cells (Figure 5E), consistent with the observation that IFNγ did not stimulate apoptosis (Figure 5A,C).

### TNF-stimulated apoptosis and permeability is effectively prevented by infliximab and adalumimab

**Figure 6:**
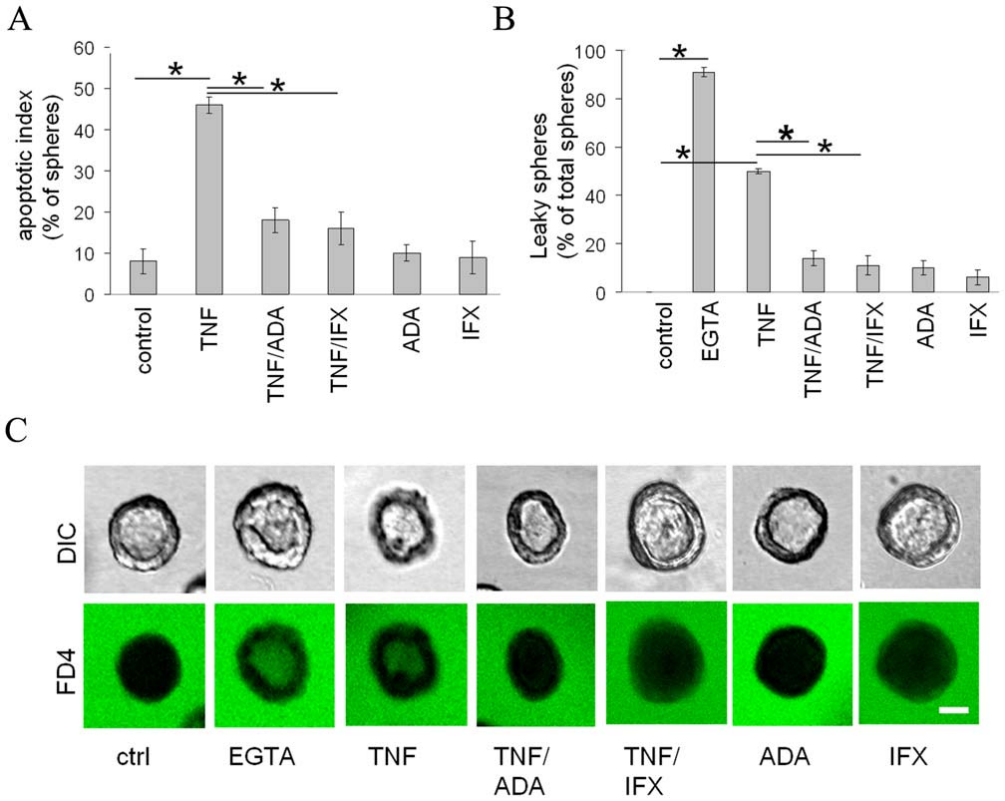
TNF-stimulated apoptosis and permeability is effectively prevented by infliximab and adalumimab. A) The apoptotic index, expressed as the percentage of all luminal spheres that contain apoptotic cells in the presence/absence of the TNF inhibitors infliximab or adalumimab is depicted in the graph. B,C) Five days-old spheres were treated or not treated with TNF and/or the TNF inhibitors infliximab or adalumimab for 48 h and exposed to FD4 at 37°C for 1 h and fixed. The percentage of luminal spheres containing apoptotic material is shown in B. Representative images are shown in C. Note that the luminal appearance of FD4 in TNF-treated spheres is counteracted by the co-incubation with either infliximab or adalumimab. Asterisks indicate statistical significance in a Student's t-test (p<0.05). Bars indicate 10 µm.

Infliximab and adalumimab are two TNF inhibitors that are used in the clinic for the treatment of inflammatory Crohn's disease. We next determined whether infliximab and adalumimab were able to prevent TNF-stimulated apoptosis and monolayer permeability. For this, 5 day-old (post-seeding) spheres were treated with or without TNF and/or infliximab or adalumimab (1 µg/ml). Treatment with either infliximab or adalumimab prevented apoptosis in TNF-treated cells, similar to cells that were cotreated with QVD (Figure 5) and resulting in spheres with virtually no luminal cellular debris (Figure 6A). We then examined the permeability of the luminal spheres by including FD4 in the medium. Whereas TNF-treated spheres showed a pronounced accumulation of FD4 in their apical lumens (Figure 6B,C), co-treatment with either infliximab or adalumimab prevented the accumulation of FD4 into the apical lumens (Figure 6B, C). Treatment with either infliximab or adalumimab in the absence of TNF was without effect (Figure 6). Together these data indicate that the presence of the TNF inhibitors infliximab or adalumimab effectively prevent TNF from stimulating apoptosis and enhancing monolayer permeability.

### TNF perturbs intestinal epithelial morphogenesis

**Figure 7:**
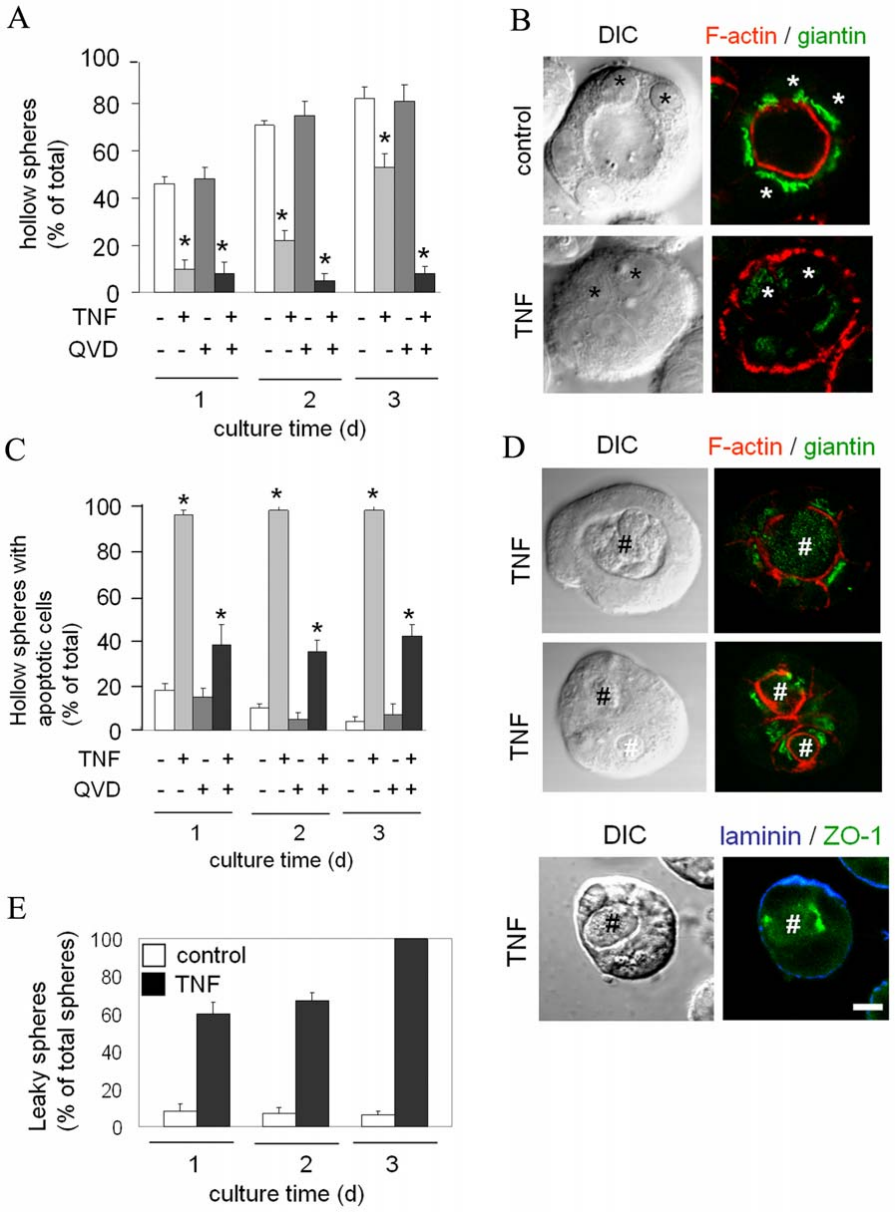
TNF interferes with 3D intestinal epithelial cell morphogenesis (T84). A) Cells were plated in Matrigel in the presence or in the absence of TNF and/or the caspase inhibitor QVD. The number of hollow spheres (expressed as percentage of all spheres) is depicted as function of time following cell plating. B) Cells were cultured for 48 h in Matrigel in the presence or absence of TNF, fixed and immunolabeled with antibodies against the Golgi-associated protein giantin (green) and stained with the actin filament-binding dye TRITC-phalloidin (red). Corresponding DIC images are depicted in the left column. Asterisks indicate position of the nucleus of two or three cells in the spheres. C) Cells were plated in Matrigel in the presence or in the absence of TNF and/or the caspase inhibitor QVD. The number of hollow spheres containing apoptotic cells (expressed as percentage of all hollow spheres) is depicted as function of time following cell plating. D) Cells were cultured for 24 h in Matrigel in the presence or absence of TNF, fixed and immunolabeled with antibodies against the Golgi-associated protein giantin (green), laminin1/2 (blue), and/or ZO-1 (green) and in some cases stained with the actin filament-binding dye TRITC-phalloidin (red). Corresponding DIC images are depicted in the left column. # indicate apoptotic cells in the sphere. E) Cells were plated in Matrigel in the presence or in the absence of TNF for 24, 48, or 72 h and exposed to FD4 at 37°C for 1 h and fixed. The percentage of total hollow spheres that contained FD4 in their lumen is depicted. Bars indicate 10 µm.

We then investigated the effect of TNF and IFNγ exposure on intestinal epithelial morphogenesis and the ability of intestinal epithelial cells to form tight monolayers in 3D culture. Cells, suspended in Matrigel, were cultured in control medium or medium supplemented with TNF or IFNγ. In control cells cultured for 24 h, lumens were detected in 46% of the spheres (Figure 7A). Comparable numbers were obtained with IFNγ-treated cells (Figure S2,A). In contrast, in TNF-treated cells, mainly solid clumps of cells with small F-actin-rich protrusions on the basal delineating membrane were observed at this time point, and only 10% of these contained a lumen (Figure 7A). Following another 24 h, the number of control (Figure 7A), and IFNγ-treated (Figure S2,A), spheres with a lumen increased to 60–70%. In TNF-treated cells only 22% of the spheres did reveal lumens (Figure 7A). After 72 h in culture, 70–80% of the control (Figure 7A), and IFNγ-treated spheres (Figure S2,A), had formed one or multiple lumens. In the presence of TNF, only 53% of the spheres had formed one or more lumens (Figure 7A). The orientation of the Golgi complex is a measure of internal cell polarity. In TNF-treated spheres lacking a lumen the Golgi complex was randomly positioned or was facing the F-actin-rich basal delineating surface (Figure 7B), which is typical for the absence of an apical-basal axis of polarity in these cells [18], and which is in striking contrast to the predominant orientation of the Golgi complex towards the F-actin-rich luminal surface of the epithelial cells in control spheres (Figure 7B).

The vast majority (98%) of lumens in 48 h-old cultures and 72 h-old T84 cultures incubated with TNF coincided with the presence of apoptotic cells. In sharp contrast, lumens in control and IFNγ-treated spheres in general did not contain apoptotic cells (<20% of all spheres, Figure 7C and Figure S2,B). In virtually all (98%) TNF-exposed spheres cultured for 72 h the lumens were filled with apoptotic material (Figure 7C, illustrated in 7D, upper row). Cells surrounding an apoptotic cell in TNF-treated clusters accumulated filamentous actin and repositioned their Golgi complex (Figure 7D, middle row) and the tight junction-associated protein ZO-1 (Figure 7D, bottom row) towards the apoptotic cell, even before a noticeable lumen could be observed. However, in contrast to non-treated spheres, all lumens in TNF-treated spheres contained FD4 when FD4 was added to the culture medium (Figure 7E), suggesting the absence of a tight junction-based paracellular barrier for small molecules. Basal laminin remained restricted to the basal surface of TNF-treated spheres (Figure 7D, bottom row). Importantly, in the presence of the caspase inhibitor QVD, virtually no lumens were formed in the presence of TNF, whereas the caspase inhibitor alone did not inhibit lumen formation (Figures 7A and 7C). These data demonstrate that exposure to TNF inhibited the efficient development of tight epithelial monolayers and lumens. As a function of time, TNF induced the formation of leaky monolayers and lumens via a mechanism that involved apoptosis, while lumen formation in the control situation typically does not involve apoptosis.

## Discussion

Here we have employed a 3D cell culture system for the study of intestinal epithelial integrity, permeability, and morphogenesis in response to the proinflammatory cytokines TNF and IFNγ. In this system, T84 and Caco-2 cells, as a function of time, formed monolayered spheres that separated a central apical lumen from the basal environment, consistent with previous reports [16], [18], [21]. Cells in such hollow spheres coordinately segregated structurally, compositionally, and functionally distinct cell surface domains resembling the *in vivo* architecture of enterocytes. These included i) an apical surface rich in microvilli, cortical F-actin and typical brush border proteins, ii) a basal surface that expressed integrin receptors and recruited the basement membrane protein laminin, and iii) a lateral surface with typical epithelial cell-cell adhesion junctions and associated proteins such as β-catenin and ZO-1. Furthermore, live cell imaging revealed that the intestinal epithelial cells in 3D culture formed a tight monolayer, impermeable to low molecular weight molecules.

Exposure of pre-assembled monolayered (i.e. luminal) T84 or Caco-2 spheres to typical Th1 cytokines TNF or IFNγ induced paracellular permeability. While the enhancement of permeability as such is consistent with previous results obtained with 2D cell culture systems, our data demonstrate clear differences between the two cytokines with regard to the kinetics of paracellular leakage as measure in real-time and the mechanisms involved. Thus, in TNF-, but not IFNγ- exposed T84 spheres, the tight junction protein occludin was displaced from the apical-most aspect of the lateral surface, where ZO-1 maintained its residence. A redistribution of claudin-1 but not of occludin or ZO-1 to intracellular punctae was observed both in TNF- and IFNγ-treated cells. Previous studies with 2D culture systems have demonstrated a more dramatic redistribution and internalization of tight junction proteins in response to TNF and IFNγ [24], [25]. It is conceivable that the interaction of the cells with the basal extracellular matrix in the 3D culture influences the dynamics of tight junction proteins (see also below).

TNF, but not IFNγ, only moderately stimulated the mitotic index and did not interfere with the orientation of cell division, which is carefully regulated and an important factor in maintaining proper intestinal epithelium architecture [15], [16]. Indeed, TNF did not visibly affect the overall architecture of the spheres. By contrast, however, TNF, but not IFNγ, significantly stimulated the accumulation of apoptotic cell debris in the apical lumens of these pre-formed epithelial spheres, which is most likely the result of apoptotic cell extrusion into the lumen. Inhibition of apoptosis with a caspase inhibitor inhibited TNF-stimulated, but not IFNγ-stimulated paracellular permeability. The role of apoptosis in enhanced intestinal paracellular permeability is debated in the literature [22], [23], [25]. The effect of TNF on tight junction function, i.e. the redistribution of tight junction proteins and/or changing the membrane/lipid properties of tight junctions, is a widely accepted mechanism via which TNF contributes to enhanced monolayer permeability (reviewed in [24]). Although we do not exclude an involvement of tight junction perturbation, possibly including the redistribution of occludin, claudin-1, and/or other tight junction proteins, in TNF- or IFNγ-stimulated paracellular permeability, our data clearly show that paracellular permeability of 3D cultured monolayers induced by TNF, but not by IFNγ, is primarily mediated by TNF-stimulated apoptosis.

The effects of TNF on apoptosis and permeability were effectively inhibited when the cells were cotreated with infliximab or adalumimab, which are mouse-human chimeric and fully human TNF-neutralizing monoclonal antibodies, respectively, that are used in the clinic for the treatment of inflammatory Crohn's disease.

The responsiveness of T84 cells to TNF and IFNγ as reported in this study contrast results obtained by Bruewer and colleagues [25]. It is conceivable that this is the result of the different treatment conditions used (i.e., 5 h TNF treatment [25] vs. 72 h TNF treatment (this study)), read-outs (i.e., TER measurement and quantitative FD3 flux [25] vs. imaging-based quantitative FD4 flux (this study)) and, importantly, the cell culture model system used (i.e., 2D [25] vs. 3D cultures (this study)). Indeed, with respect to the latter, it is not unprecedented that cells in 2D or 3D culture respond differently to stimuli. Hepatic epithelial cells, for instance, display exacerbated NFkB signalling and cell survival when cultured in a monolayer, whereas their culture in a 3D matrix sensitizes them to apoptotic signalling [26]. Also Madin-Darby canine kidney cells in 2D or 3D culture respond differently to transformation by v-Src [27]. Collectively, these results thus emphasize the importance of the 3D cell culture model system.

3D cell culture systems particularly provide an opportune model system and are increasingly used for the study of epithelial morphogenesis. Characteristic for (single-layered) epithelial morphogenesis is the development of internal and cell surface polarity and the assembly of individual cells into a monolayer that physically separates an apical and basal extracellular environment. Two main mechanisms for epithelial monolayer development have emerged [13], [19], in which the extracellular matrix plays an important role. Studies in renal tubular [19], [28], [29] and intestinal [16] epithelial cells suggest that extracellular matrix-RhoGTPase-elicited signalling pathways cause individual cells to establish an axis of polarity and organize themselves such that the secretion of fluid-filled and apical protein-carrying transport vesicles occurs in a coordinated and polarized fashion. This produces an apical surface-lined intercellular cavity that is typically positioned opposite to the extracellular matrix-facing basal surface. Subsequent cell divisions that are strictly orientated perpendicular to the newly formed apical surface help expand the monolayer and apical lumen. By contrast, other epithelia, including mammary [30] and salivary gland epithelia [31], develop single-layered lumens via an alternative mechanism. Here, extracellular matrix surrounding a cluster of cells promotes the selective survival of those cells that are in direct contact with the matrix. Cells in the centre of the clump die through apoptosis, creating an apical lumen surrounded by a single layer of matrix-facing epithelial cells. Such monolayers typically display enhanced paracellular permeability [32]. Of interest, when renal tubular epithelial cells fail to respond to the polarization cue from the extracellular matrix, these can form monolayers via the latter alternative mechanism by virtue of the natural occurrence of apoptosis [33]. Apoptosis thus can provide for a backup mechanism for epithelial monolayer development during epithelial morphogenesis when the acquisition of apical-basal polarity is disrupted or delayed [34].

**Figure 8:**
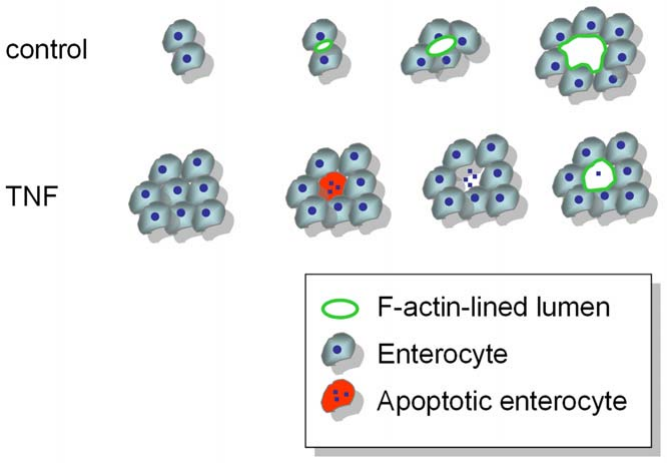
Cartoon illustrating the working model of apical lumen and monolayer formation in control and TNF-treated cells. Non-treated cells develop single apical lumens and expanding monolayers with no correlation to apoptosis. In contrast, TNF-treated cells at first form solid cysts, followed by apoptosis of single cells within the cyst and polarization of the surrounding cells.

We show that under control conditions, intestinal epithelial T84 and Caco-2 cells developed polarity and self-organized into tight luminal spheres separating apical and basal environments. Whereas Caco-2 cells developed heterogeneous luminal spheres, which prohibited an accurate examination of morphogenesis, T84 cells developed uniform luminal spheres with no correlation to the presence of apoptotic material. Together with the observed orientation of T84 cell divisions, our data are consistent with the mode of epithelial morphogenesis reported for intestinal epithelial cells [16]. IFNγ did not inhibit luminal sphere formation but an increasing number of formed spheres displayed paracellular leakage as a function of time. In contrast to control and IFNγ-exposed cells, exposure of T84 cells to TNF inhibited the initial development of an apical-basal polarity axis. Nevertheless luminal spheres were formed, however with a severe delay and to a reduced extent when compared to controls. The vast majority of these luminal spheres displayed high paracellular permeability without visible changes in the typical basal localization of laminin. In strong contrast to control and IFNγ-treated cells, the majority of lumens in the monolayer-lined spheres that were formed in the presence of TNF, from the earliest time points observed, correlated with the presence of apoptotic cells. Furthermore, co-treatment of the cells with an inhibitor of caspase activity, was shown to prevent TNF-induced apoptosis, precluded luminal sphere formation. These data suggest that TNF inhibited polarization and lumen formation by T84 cells in response to extracellular matrix stimuli. Which of the emerging molecular players involved in lumen formation [34], [35] is targeted by TNF remains to be investigated. TNF, by stimulating apoptosis, however, allowed polarization of cells that surround the dying cell and the formation of monolayers and lumens, albeit leaky, via this alternative mechanism. An illustrating cartoon is depicted in figure 8. It is important to note that, in contrast to mammary and saliva epithelium in which apoptosis appears to be a process that is tightly regulated by the interaction of the cells with the surrounding matrix, TNF-stimulated apoptosis in intestinal epithelial cells was not restricted to centrally located cells. The random occurrence of apoptosis in TNF-treated spheres can explain the delay and/or limited extent by which luminal spheres were formed.

Our data demonstrate that the proinflammatory cytokine TNF inhibits normal intestinal epithelial morphogenesis, which would lead to the formation of tight epithelial monolayers and instead, by stimulating apoptosis, promotes an alternate mode of gut epithelial morphogenesis, resulting in the formation of ‘leaky’ monolayers. Once tight intestinal epithelial monolayers are formed, exposure to TNF or IFNγ does not perturb their overall monolayer organization, but increases paracellular permeability with apoptosis as an important mediator for TNF, but not for IFNγ. On a cautionary note, whereas the stimulatory effects of TNF on apoptosis and permeability were demonstrated in both T84 and the single lumen-containing Caco-2 cells, the heterogeneity of Caco-2 sphere development allowed demonstration of TNF effects on epithelial morphogenesis in T84 cells only. Further optimalization of the 3D culture for Caco-2 or other epithelial cells are awaited to further substantiate the effects of TNF on intestinal epithelial morphogenesis as reported in this study. Similar results were obtained when growth factor-reduced Matrigel (BD Bioscience #356230) was used instead of normal Matrigel (our unpublished data), indicating that the effects of TNF are not modulated by matrigel-derived growth factors. We postulate that TNF contributes to a reduced mucosal and epithelial integrity as observed in inflammatory bowel diseases by perturbing and altering the mode of intestinal epithelial morphogenesis and, in this way, the functional differentiation of regenerative intestinal mucosa.

## Supporting Information

Figure S1 **Caco-2 cells in 3D culture develop a polarized, yet heterogenous spheres.** A) Low magnification phase contrast image of 7 days-old Caco-2 in 3D culture. B) gp135 (podocalyxin) and ZO-1 are exclusively expressed at the actin filament-rich luminal cell surface of the spheres, whereas the β-1 integrin receptor localizes to the basolateral domain of the luminal sphere formed by Caco-2 cells. C) Effect of EGTA on paracellular permeability in 3D Caco-2 lumenal spheres. Cultures were treated or not treated with EGTA and exposed to FD4 at 37°C for 1 h and fixed. FD4 is in the apical lumens EGTA-exposed, but not untreated spheres (left column), corresponding DIC images are in the middle column, and merged images are in the right column.
(TIF)

Figure S2 **IFNγ has no effect on 3D intestinal epithelial cell morphogenesis.** A) Cells were plated in Matrigel in the presence or in the absence of IFNγ. The number of luminal spheres (expressed as percentage of all spheres) is depicted as function of time following cell plating. B) Cells were plated in Matrigel in the presence or in the absence of IFNγ The number of hollow spheres containing apoptotic cells (expressed as percentage of all luminal spheres) is depicted as function of time following cell plating. C) Cells were plated in Matrigel in the presence or in the absence of IFNγ for 24, 48, or 72 h and exposed to FD4 at 37°C for 1 h and fixed. The percentage of total luminal spheres that contained FD4 in their lumen is depicted.
(TIF)

Movie S1 **Three-dimensional reconstruction of a T84 sphere.** Cells were cultured for 7 days in matrigel to form hollow spheres. Cells were processed for immunolabeling with antibodies against the adherens and tight junction proteins β-catenin (in red) and ZO-1 (in green), respectively. Confocal stacks (over sampled) were generated, deconvoluted, 3D reconstructed as described in Materials and Methods. An animation of one 3D reconstructed luminal T84 sphere is shown.
(MPG)
