# Supplementary figures and images for: Differential Effects of TNF (TNFSF2) and IFN-γ on Intestinal Epithelial Cell Morphogenesis and Barrier Function in Three-Dimensional Culture
Source: PLoS One. 2011 Aug 11;6(8):e22967. doi: 10.1371/journal.pone.0022967 (PMC3154921; doi:10.1371/journal.pone.0022967)

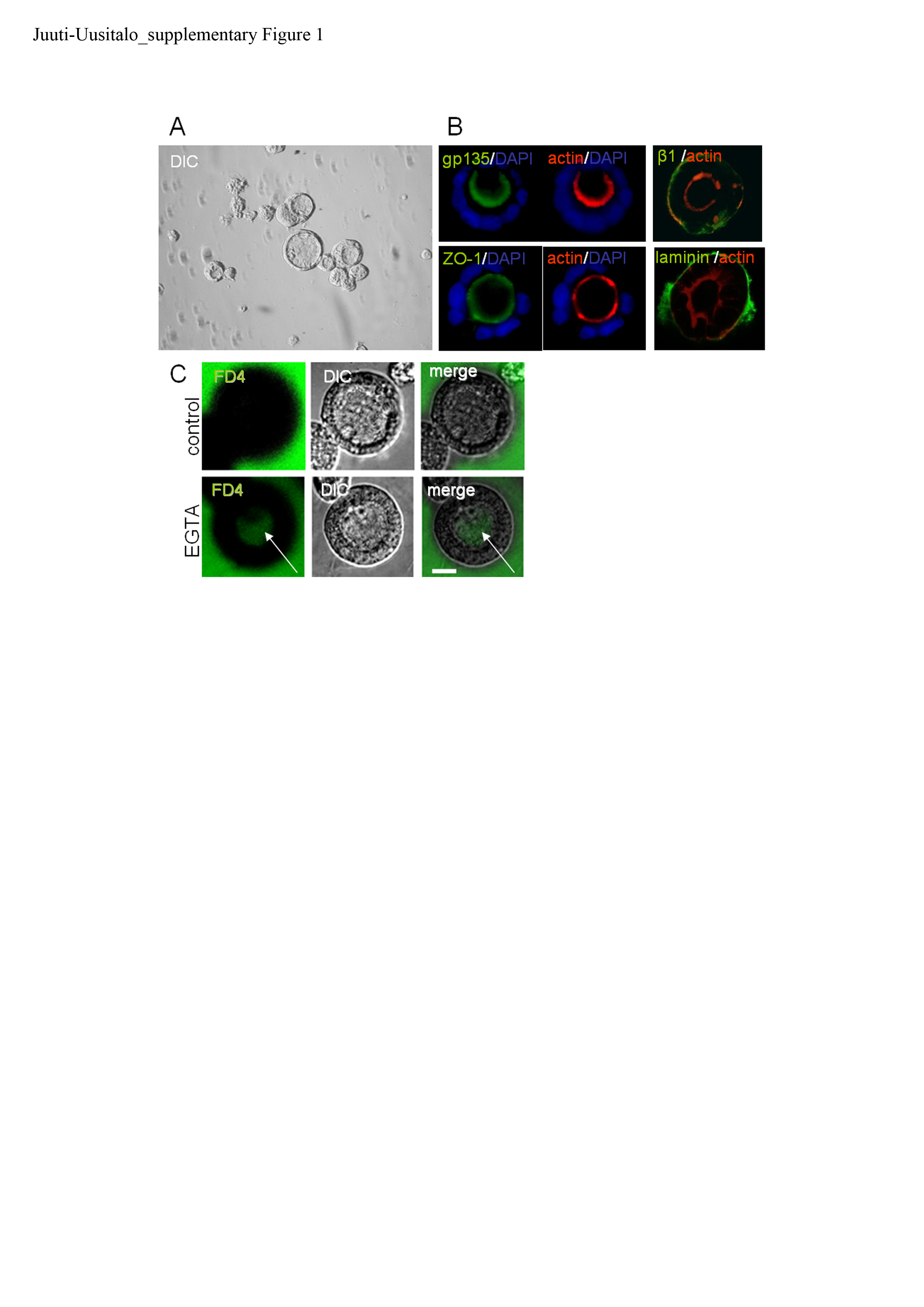

Supplement: Figure S1 — Caco-2 cells in 3D culture develop a polarized, yet heterogenous spheres. A) Low magnification phase contrast image of 7 days-old Caco-2 in 3D culture. B) gp135 (podocalyxin) and ZO-1 are exclusively expressed at the actin filament-rich luminal cell surface of the spheres, whereas the β-1 integrin receptor localizes to the basolateral domain of the luminal sphere formed by Caco-2 cells. C) Effect of EGTA on paracellular permeability in 3D Caco-2 lumenal spheres. Cultures were treated or not treated with EGTA and exposed to FD4 at 37°C for 1 h and fixed. FD4 is in the apical lumens EGTA-exposed, but not untreated spheres (left column), corresponding DIC images are in the middle column, and merged images are in the right column. (TIF) [file pone.0022967.s001.tif]

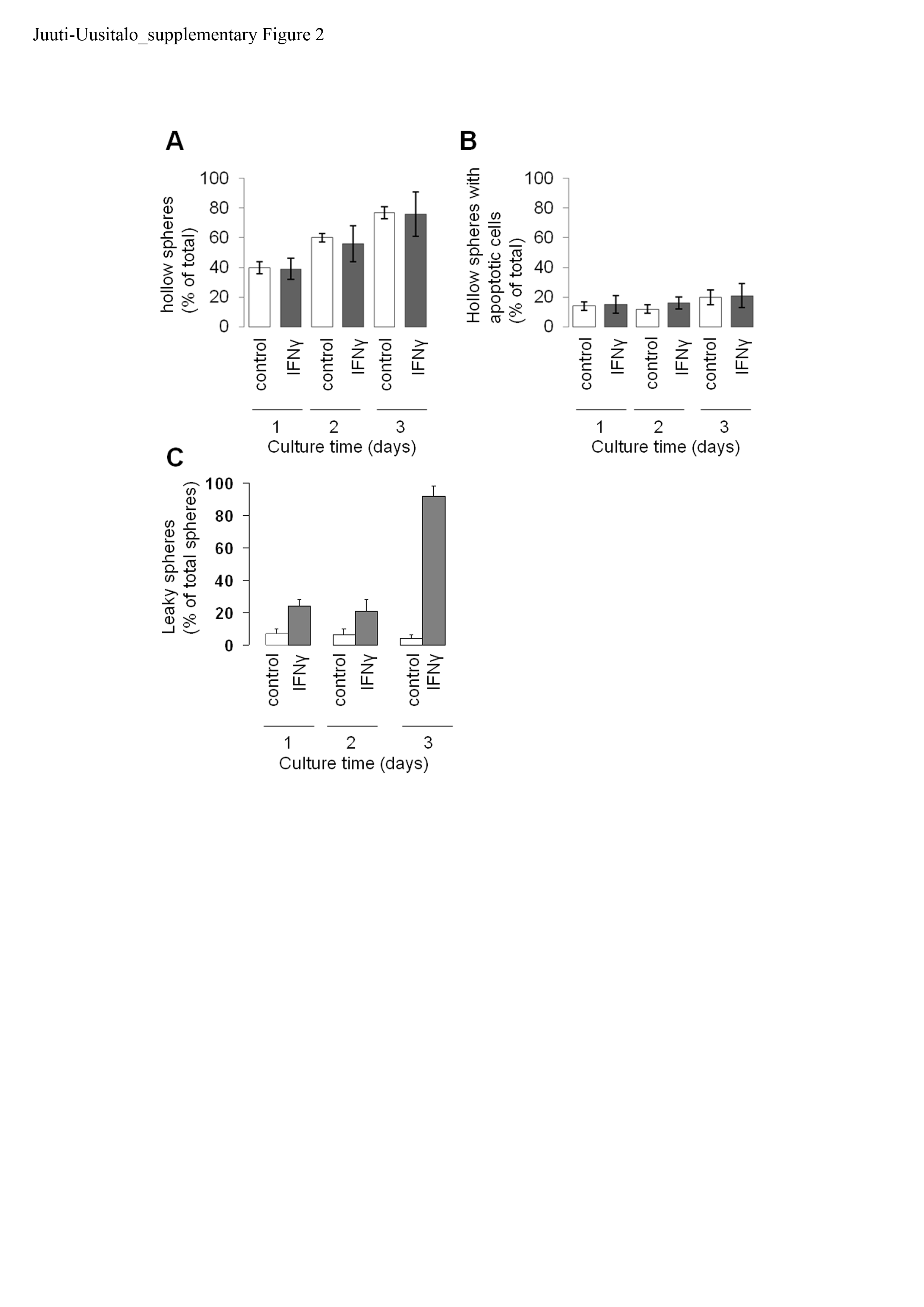

Supplement: Figure S2 — IFNγ has no effect on 3D intestinal epithelial cell morphogenesis. A) Cells were plated in Matrigel in the presence or in the absence of IFNγ. The number of luminal spheres (expressed as percentage of all spheres) is depicted as function of time following cell plating. B) Cells were plated in Matrigel in the presence or in the absence of IFNγ The number of hollow spheres containing apoptotic cells (expressed as percentage of all luminal spheres) is depicted as function of time following cell plating. C) Cells were plated in Matrigel in the presence or in the absence of IFNγ for 24, 48, or 72 h and exposed to FD4 at 37°C for 1 h and fixed. The percentage of total luminal spheres that contained FD4 in their lumen is depicted. (TIF) [file pone.0022967.s002.tif]
